# Association between various physical activity domains and overall cancer risk, National Health and Nutrition Examination Survey (NHANES) 2007–2018

**DOI:** 10.1371/journal.pone.0308099

**Published:** 2024-07-29

**Authors:** Yanxue Lian, Pincheng Luo

**Affiliations:** Department of Physiology, School of Medicine, University of Galway, Galway, Ireland; University of Essex, UNITED KINGDOM OF GREAT BRITAIN AND NORTHERN IRELAND

## Abstract

**Purpose:**

There are very few studies concurrently evaluating the association between multiple physical activity (PA) domains and cancer prevalence. Therefore, this study aims to fill this gap by investigating the link between multiple PA subdomains [occupational PA (OPA), transportation-related PA (TPA), leisure-time PA (LTPA), and total PA] and the likelihood of cancer.

**Method:**

The data from National Health and Nutrition Examination Survey (NHANES) 2007–2008, 2009–2010, 2011–2012, 2013–2014, 2015–2016, and 2017–2018 were used in this study. Cancers are the primary outcome variable of interest in this study. PA was self- or proxy-reported using the Global Physical Activity Questionnaire (GPAQ). Multivariable logistic regression models were used, adjusted for covariates.

**Results:**

The trend analysis revealed that the prevalence of cancer statistically decreased with the increase in total PA amount. The participants achieving twice the minimum recommended PA guidelines (≥300 minutes) for total PA were 32% [0.68 (0.54, 0.86)] less likely to have cancer. However, significant associations between three PA subdomains (OPA, TPA, and LTPA) and cancers were not found in this study.

**Conclusion:**

There is no significant association between any of these three single PA subdomains and cancer prevalence other than total PA. Therefore, this study recommends clinical practice should prioritize promoting comprehensive PA that integrates OPA, TPA, and LTPA to achieve at least 150 minutes per week (i.e. per seven days) initially and progressing towards 300 minutes for optimal cancer prevention.

## 1. Introduction

Cancer, characterized by the uncontrolled growth and spread of abnormal cells in the body, can originate from almost any part of the body [1]. There are more than one hundred cancer types according to National Cancer Institute (NCI) [1]. People suffering from cancer may experience numerous symptoms, such as pain, fatigue, dyspnea, abnormal bleeding, and abnormal weight loss [2, 3]. These symptoms can cause impaired physical fitness, reduced quality of life, and more seriously, death [3]. Over 1.9 million new cancer cases were reported with 609,820 deaths from cancer (1,670 deaths per day) in America in 2023, which does not include the cases of certain cancer types, such as basal cell and squamous cell skin cancers [4]. Therefore, effective strategies are critical for prevention and treatment of cancer. Some studies show that not only medical approaches but also lifestyle factors are associated with cancer incidence or cancer-related mortality, and these lifestyle factors include smoking, alcohol consumption, diet, body weight, and physical activity (PA) [5, 6].

PA refers to any body movement produced by the musculoskeletal system, which can cause energy expenditure [7]. PA consists of different domains: occupational PA (OPA) occurring in any working environment [8], transportation-related PA (TPA) defined as healthy active travel behaviors during commuting [9], and leisure-time PA (LTPA) performed during exercise or recreation or any time that is not related to one’s regular occupation, housework, or transportation [10]. Distinct PA domains have been shown to exert a significant influence on overall health outcomes, potentially affecting a range of chronic diseases, such as stroke [11, 12], cardiovascular diseases [13, 14], and diabetes [15, 16]. Additionally, some studies have investigated the association between total PA and cancers [17–21], while some studies have focused on a single PA subdomain and cancers [22–24]. However, there are very few studies concurrently evaluating the association between multiple PA domains and cancer prevalence.

Therefore, this study aims to fill this gap by investigating the link between multiple PA subdomains (OPA, TPA, LTPA, and total PA) and the likelihood of cancer. To make OPA and TPA more relevant, the emphasis is on individuals under 65, since they are generally more active in the workforce and daily commuting [25]. Focusing on a younger population helps understand preventive measures and early interventions that could potentially reduce cancer risk before the typical increase in incidence seen in older adults. Moreover, whether the association between these PA domains and cancer would be altered by other factors will also be examined. The dose-response association between the related PA domains and cancer will be further assessed. Moreover, a Receiver Operating Characteristic (ROC) curve will be constructed to assess the accuracy of predicting cancer using the amount of total PA.

## 2. Materials and method

### 2.1. Participants

**Fig 1:**
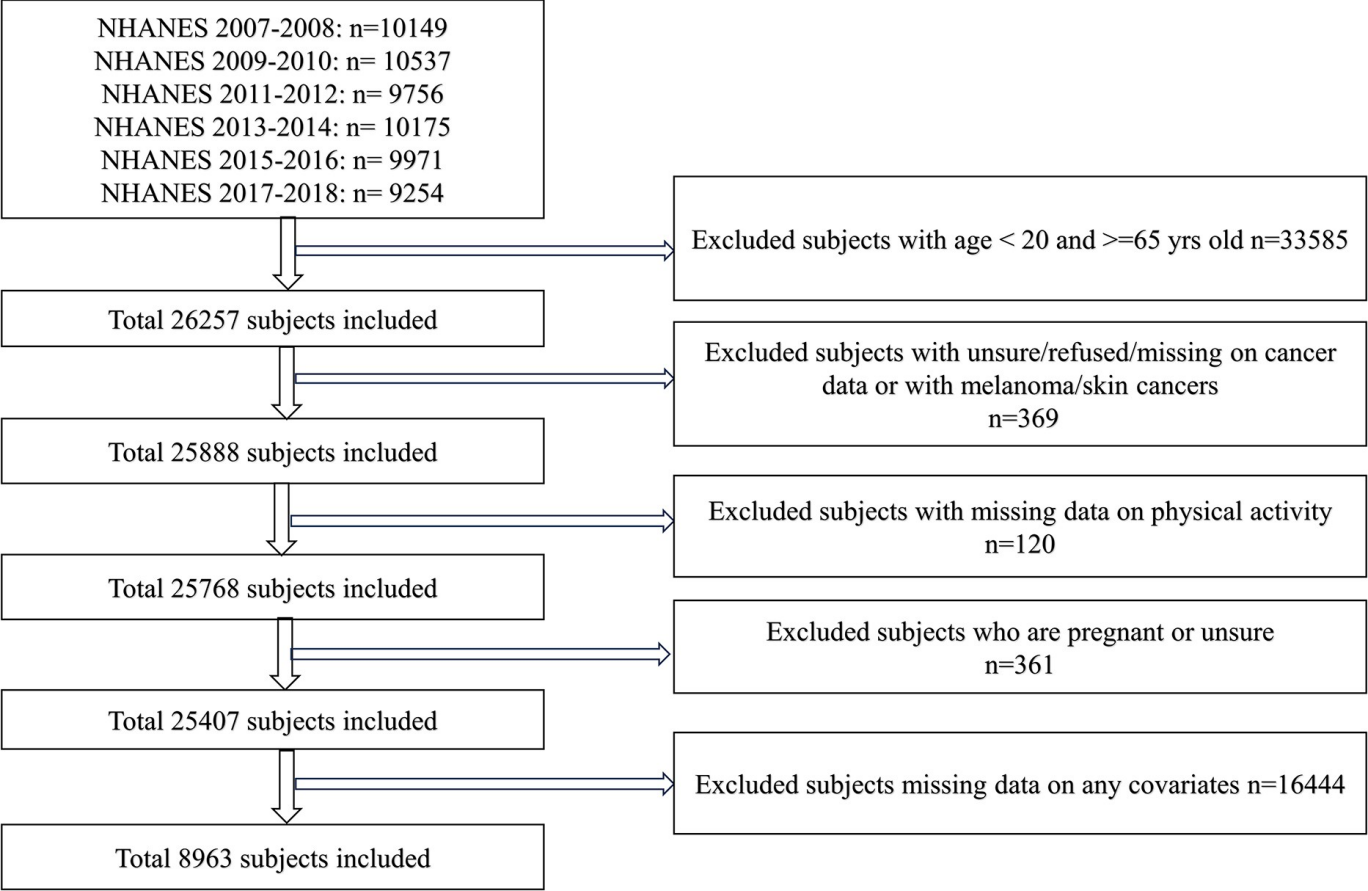
Flow chart of the participants’ selection. Abbreviation: NHANES, National Health and Nutrition Examination Survey.

The National Health and Nutrition Examination Survey (NHANES) (https://www.cdc.gov/nchs/nhanes/, accessed on the 4^th^ of April, 2024 for research purposes), which mainly consists of interviews and examinations, is a continuous program conducted every two years to representatively examine the health and nutrition status of U.S. residents. In compliance with ethical standards and to ensure participant confidentiality, no identifying information was accessible during or after data collection. The data from NHANES 2007–2008, 2009–2010, 2011–2012, 2013–2014, 2015–2016, and 2017–2018 were used in this study, focusing on a population that is actively engaged in work and commuting activities. Therefore, participants were not included if they were <20 or ≥65 years old. Additionally, they were excluded if they had melanoma/skin cancers (non-melanoma)/skin cancers (don’t know what kind), with any missing data on PA, were currently pregnant/unsure if pregnant, or with missing data (including refused, and don’t know) on any covariates. The final analytic population included 8936 subjects (**Fig 1**). The survey has been approved by the National Center for Health Statistics Research Ethics Review Board, and consent forms were obtained from all subjects. This study was performed as a secondary analysis, so no additional ethical approval was needed.

### 2.2. Outcome

Cancers are the primary outcome variable of interest in this study. Cancer survivors were identified based on self- or proxy-reported medical conditions questionnaires (MCQ), if the answer was “Yes” to the question “{Have you/Has SP} ever been told by a doctor or other health professional that {you/s/he} had cancer or a malignancy (ma-lig-nan-see) of any kind? (questionnaire code: MCQ220)” If the answer was “No”, participants were categorized into the non-cancer group. Subjects who had melanoma/skin cancers (non-melanoma)/skin cancers (don’t know what kind) were not considered.

### 2.3. Physical activity

PA was self- or proxy-reported using the Global Physical Activity Questionnaire (GPAQ). This is a validated instrument developed by the World Health Organization (WHO) for assessing PA levels in adults aged 18–65 years across different domains: OPA, LTPA, and TPA [26]. GPAQ collects information on frequency (number of days per typical week, defined as 7 days) and duration (minutes in a typical day) in all three subdomains of PA, as well as the intensity (vigorous or moderate) of OPA and LTPA [26]. The questionnaire provides a comprehensive assessment of PA behaviors, facilitating comparisons across populations and studies. Some previous studies suggest that 1 minute of vigorous-intensity PA can be simply converted into 2 minutes of moderate-intensity PA for easy calculation in terms of energy consumption [27, 28]. In other words, the minutes spent on OPA/LTPA can be calculated by doubling the minutes of vigorous-intensity PA, plus the minutes of moderate-intensity PA. Thus, the minutes engaged in OPA, LTPA, and TPA in a typical week were calculated. Additionally, the time of total PA refers to the sum of the time of OPA, LTPA, and TPA.

According to the WHO 2020 guidelines on PA, adults including those with chronic conditions are encouraged to engage in at least 150–300 minutes of moderate-intensity PA, or 75–150 minutes of vigorous-intensity PA, or some equivalent combination of both [29]. Participants were divided into two groups: (1) participants who met the minimum requirements of the WHO 2020 guidelines on PA (the minutes of PA ≥ 150 in a typical week), and (2) participants who did not meet the minimum requirements of the WHO 2020 guidelines on PA (the minutes of PA < 150 in a typical week). Subsequently, based on their time of PA per week, participants were further divided into 4 groups: (1) 0 minutes, (2) 1–149 minutes, (3) 150–299 minutes, and (4) ≥ 300 minutes.

### 2.4. Covariates

In reference to previous studies [30, 31], eight covariates of demographics and lifestyles were included. Demographic-related covariates consisted of age, gender (male and female), Body Mass Index (BMI), race (Mexican American, Non-Hispanic White, Non-Hispanic Black, and others), education (less than 9th grade, 9-11^th^ grade, high school graduates or equivalent), and marital status (accompanied and alone). Lifestyles included smoking (everyday, somedays, and not at all), and alcohol consumption (yes and no).

### 2.5. Statistical analysis

Participants’ characteristics were expressed as mean with standard deviation (SD) (continuous variables), or frequency (%) (categorical variables). Group differences were analyzed by Student’s t-test or ANOVA. To assess the association between the time of PA and cancer prevalence, three multivariable logistic regression models were used, adjusted for covariates. The results were presented as odds ratios (OR) and 95% confidence intervals (CI). A crude model aimed to initially explore the unadjusted relationship between PA duration and cancer prevalence, providing a baseline understanding of their association. Model I was adjusted for age and gender—known influential factors in PA engagement and cancer risk—to mitigate potential confounding effects and refine the analysis. Finally, Model II incorporated additional covariates beyond age and gender, including BMI, race, education level, marital status, smoking status, and alcohol consumption, aiming to comprehensively adjust for potential confounders and explore the robustness of our findings across multiple dimensions of adjustment. This hierarchical approach allowed for a nuanced examination of how various levels of adjustment influence the observed association between PA duration and cancer prevalence, enhancing the validity and reliability of the study’s conclusions. Additionally, a stratified analysis and an interaction test were conducted by using Model II to evaluate the association between the amount of PA and cancer prevalence across various strata. All tests with p-values < 0.05 were considered as significant. Furthermore, a prediction model was developed to estimate the likelihood of cancer occurrence for individuals based on the minutes of PA. R software (version 4.2.2) and EmpowerStats (version 4.2) (https://www.empowerstats.net/) were used to perform all analyses.

## 3. Results

A total of 8963 participants (4897 males and 4066 females) from NHANES 2007–2018 were included in the study, and their mean age was 44.15 (12.85) years old. Of these participants, 95.21% participants did not have any kind of cancer (non-cancer group) while 4.79% of participants were diagnosed with at least one of more than 26 types of cancer (cancer group) before the survey. The characteristics of the study population according to whether they had cancer are depicted in **Table 1**. Compared with the non-cancer group, the cancer group was significantly older; had a lower percentage of Mexican Americans but a higher percentage of non-Hispanic White; and had a larger percentage of non-smokers and non-alcohol drinkers. Moreover, the percentage of participants who met the minimum recommended minutes of PA per week was significantly higher in the non-cancer group than in the cancer group across all three PA subdomains and total PA. The logistic regression analysis of the association between meeting the PA guidelines for every PA domain and the likelihood of cancer occurrence is presented in **Table 2**. In the crude model, OPA, TPA, LTPA, and total PA that met the minimum recommended weekly minutes of PA had an inverse association with the prevalence of cancer. However, after adjusting for age and gender in model I, participants who met PA guidelines for total PA had 20% lower odds of having cancer [0.80 (0.66, 0.98)]. This negative association between total PA [0.79 (0.65, 0.97)] and cancer remained in Model II, which was further adjusted for other covariates (BMI, race, education, marital status, smoking, and alcohol consumption. However, the association of the 3 subdomains of PA (OPA, TPA, and LTPA) and cancer occurrence was not shown after adjusting for covariates in Models I and II.

**Table 1:** Characteristics of the participants.

	All	Non-cancer	Cancer	p-value
Sample, n (%)	8963	8534 (95.21)	429 (4.79)	
Age, years, mean (SD)	44.15 (12.85)	43.76 (12.83)	51.77 (10.56)	< 0.001
BMI, m/kg2, mean (SD)	29.34 (7.20)	29.34 (7.17)	29.31 (7.67)	0.93
Gender, n (%)				0.13
Male	4897 (54.64)	4678 (54.82)	219 (51.05)	
Female	4066 (45.36)	3856 (45.18)	210 (48.95)	
Education				0.47
Less than 9th Grade	679 (7.58)	644 (7.55)	35 (8.16)	
9-11^th^ Grade	1619 (18.06)	1551 (18.17)	68 (15.85)	
High school graduates or equivalent	2438 (27.20)	2327 (27.27)	111 (25.87)	
College and above	4227 (47.16)	4012 (47.01)	215 (50.12)	
Race				< 0.001
Mexican American	1248 (13.92)	1215 (14.24)	33 (7.69)	
Non-Hispanic White	4107 (45.82)	3878 (45.44)	229 (53.38)	
Non-Hispanic Black	1911 (21.33)	1815 (21.27)	96 (22.38)	
Others	1697 (18.93)	1626 (19.05)	71 (16.55)	
Marital status, n (%)				0.33
Alone	3767 (42.03)	3577 (41.91)	190 (44.29)	
Accompanied	5196 (57.97)	4957 (58.09)	239 (55.71)	
Smoking				0.02
Everyday	4010 (44.74)	3820 (44.76)	190 (44.29)	
Somedays	989 (11.03)	958 (11.22)	31 (7.23)	
Not at all	3964 (44.23)	3756 (44.01)	208 (48.48)	
Alcohol consumption				< 0.001
Yes	7310 (81.56)	7008 (82.12)	302 (70.40)	
No	1653 (18.44)	1526 (17.88)	127 (29.60)	
OPA; n (%)				< 0.001
Achieved	3876 (43.24)	3725 (43.65)	151 (35.20)	
Not achieved	5087 (56.76)	4809 (56.35)	278 (64.80)	
TPA; n (%)				0.06
Achieved	1418 (15.82)	1364 (15.98)	54 (12.59)	
Not achieved	7545 (84.18)	7170 (84.02)	375 (87.41)	
LTPA; n (%)				0.049
Achieved	2853 (31.83)	2735 (32.05)	118 (27.51)	
Not achieved	6110 (68.17)	5799 (67.95)	311 (72.49)	
Total PA; n (%)				< 0.001
Achieved	5917 (66.02)	5679 (66.55)	238 (55.48)	
Not achieved	3046 (33.98)	2855 (33.45)	191 (44.52)	

Abbreviations: SD, standard deviation; BMI, Body Mass Index; PA, physical activity; LTPA, leisure-time PA; OPA, occupation-related PA; TPA, transportation-related PA.

**Table 2:** Association between various PA domains and cancer.

	Crude model ^a^		Model I ^b^		Model II ^c^	
	OR (%95 CI)	p-value	OR (%95 CI)	p-value	OR (%95 CI)	p-value
OPA						
No	1 (Ref)		1 (Ref)		1 (Ref)	
Yes	0.70 (0.57, 0.86)	0.0006	0.85 (0.69, 1.04)	0.12	0.83 (0.67, 1.02)	0.08
TPA						
No	1 (Ref)		1 (Ref)		1 (Ref)	
Yes	0.76 (0.57, 1.01)	0.06	0.85 (0.63, 1.14)	0.28	0.86 (0.64, 1.16)	0.33
LTPA						
No	1 (Ref)		1 (Ref)		1 (Ref)	
Yes	0.80 (0.65, 1.00)	0.05	1.03 (0.83, 1.29)	0.77	1.04 (0.83, 1.31)	0.72
Total PA						
No	1 (Ref)		1 (Ref)		1 (Ref)	
Yes	0.63 (0.52, 0.76)	<0.0001	0.80 (0.66, 00.98)	0.03	0.79 (0.65, 0.97)	0.03

Note: a, without any adjustment; b, adjusted for age and gender; c, adjusted for BMI (kg/m2), education, marital status, race, smoking, and alcohol consumption in addition to Model I. Abbreviations: OR, odds ratio; CI, confidence interval; PA, physical activity; LTPA, leisure-time PA; OPA, occupation-related PA; TPA, transportation-related PA; Ref, reference.

**Fig 2:**
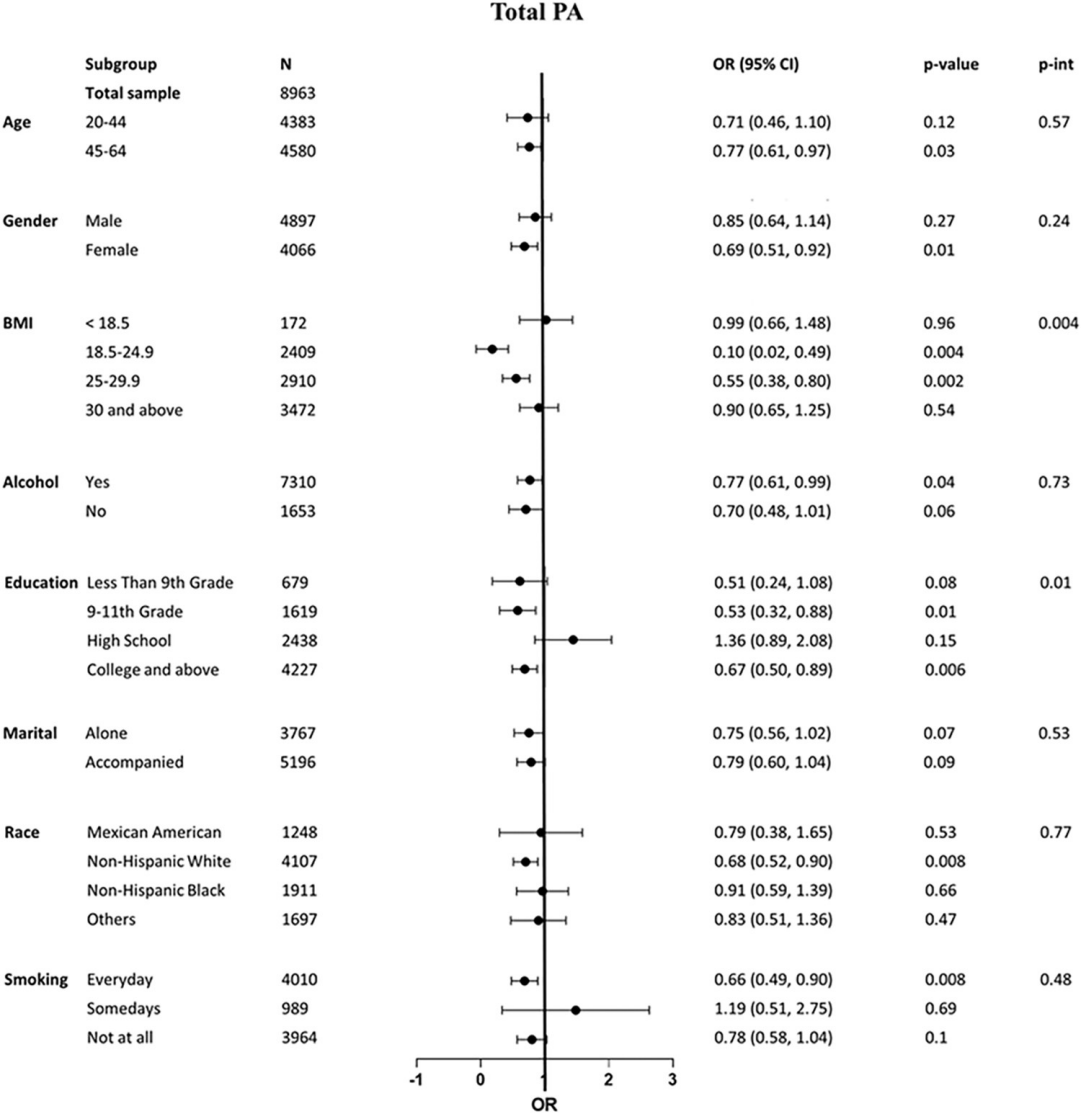
Association between total PA based on meeting the PA guidelines and cancer, stratified by 8 covariates. P-int represents the heterogeneity between subgroups based on the stratified analysis. Abbreviations: BMI, Body Mass Index; PA, physical activity; OR, odds ratio; CI, confidence interval.

Additionally, **Fig 2** shows the results of the stratified analysis and the interactions test. The stratified analysis showed that the link between the amount of total PA and cancer occurrence was not consistent although a negative relationship occurred in all categories, except for in the high school (education) and somedays (smoking) categories. The interactions test revealed that BMI and education significantly modified the association between the minutes of PA and the likelihood of cancer occurrence (p-int < 0.05). The reverse association between meeting the minimum recommended weekly minutes of PA for total PA and the likelihood of cancer occurrence was strong and significant in participants with a BMI of 18.5–24.9 kg/m2 [0.1 (0.02, 0.49)] and 25–29.9 kg/m2 [0.55 (0.38, 0.80)], but not significant in those with BMI < 18.5 or ≥ 30 kg/m2. Furthermore, this association was statistically significant in participants with an education level of 9-11^th^ grade [0.53 (0.32, 0.88)], and college and above [0.67 (0.50, 0.89)], but not in those with an education level of less than 9^th^ grade [0.51 (0.24, 1.08)] and high school graduates [1.36 (0.89, 2.08)].

**Fig 3:**
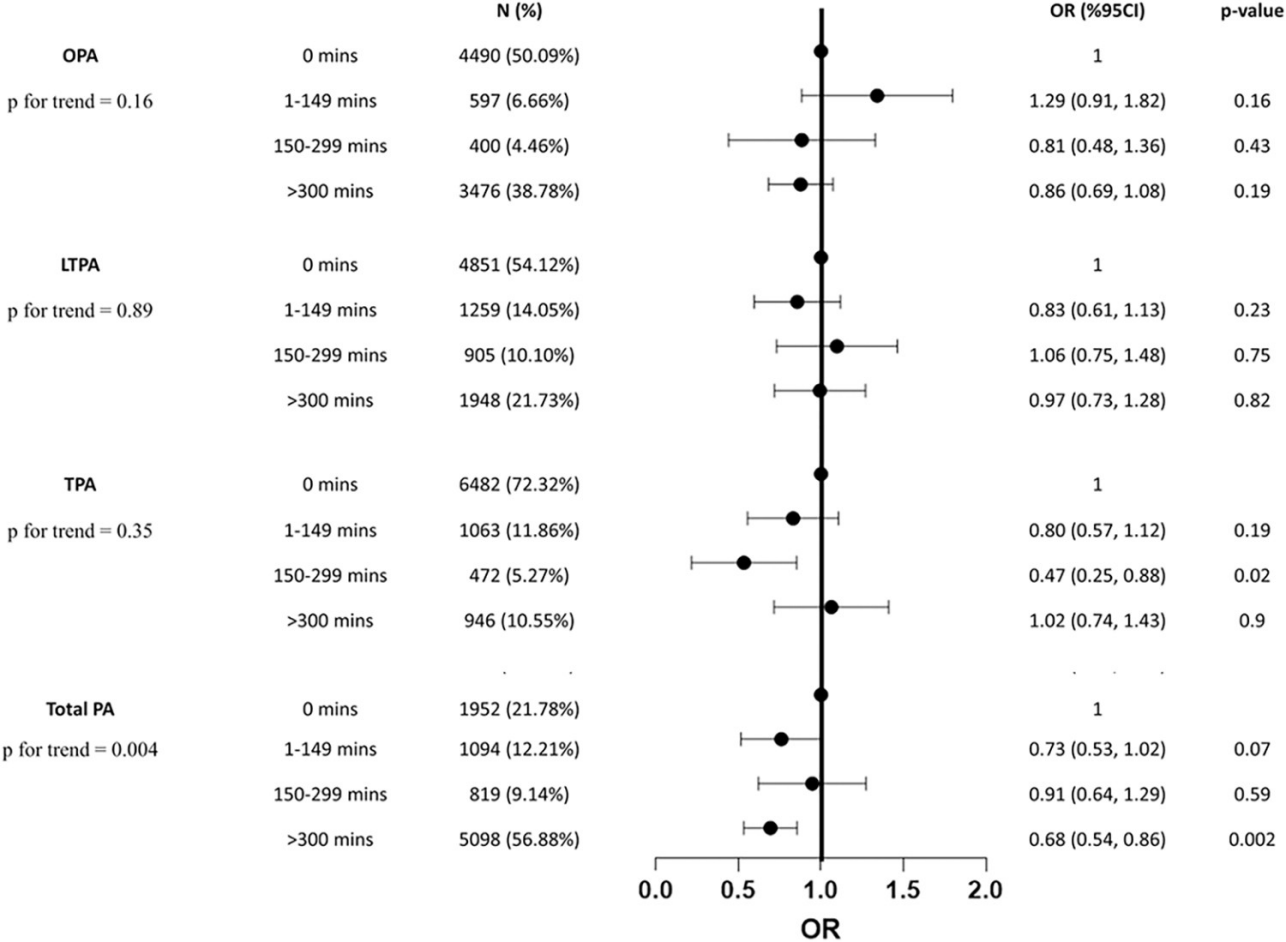
Association between various PA domains based on the amount of PA and cancer. Abbreviations: N, number; OR, odds ratio; CI, confidence interval; PA, physical activity; LTPA, leisure-time PA; OPA, occupation-related PA; TPA, transportation-related PA.

**Fig 4:**
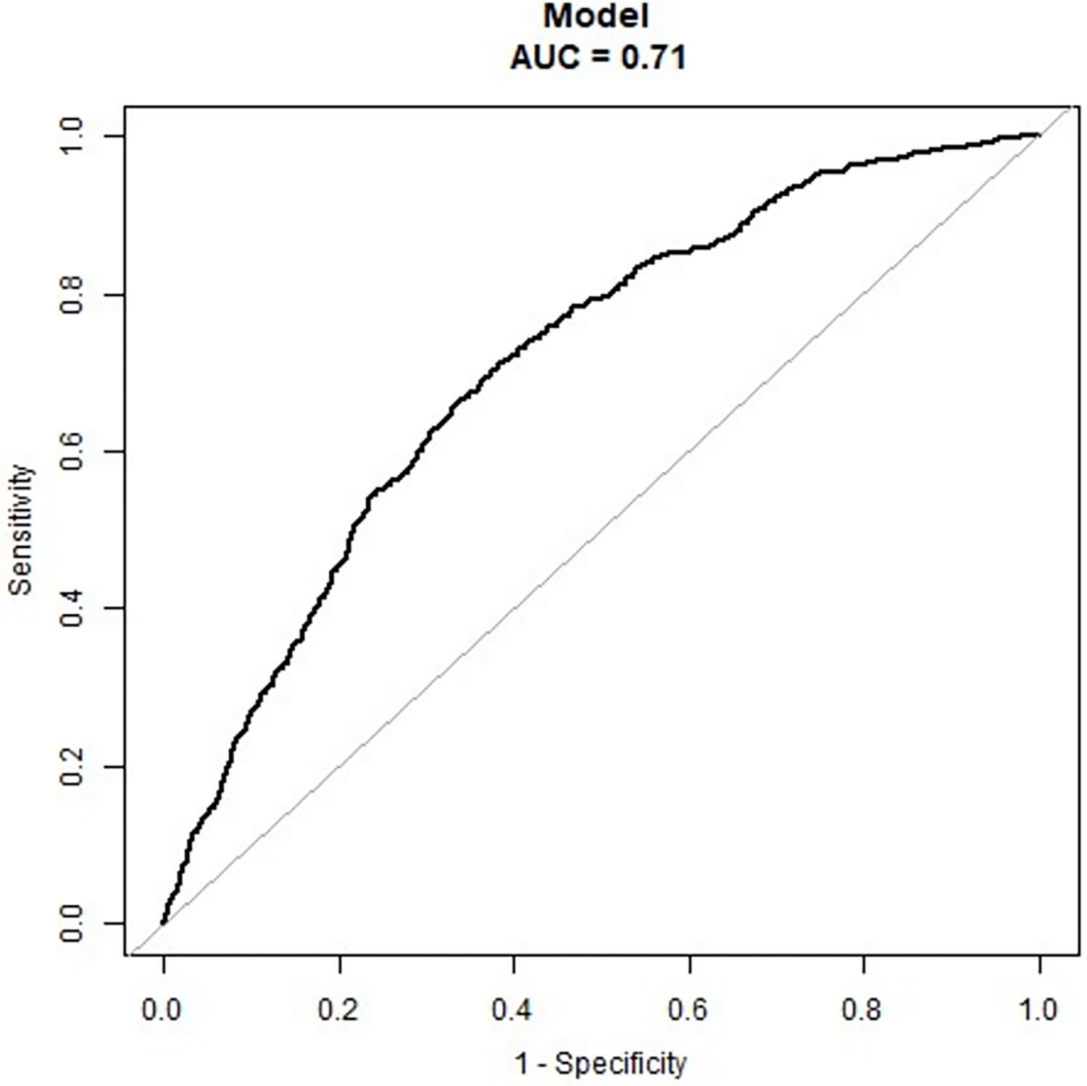
The receive operate curve of the prediction model. Abbreviations: AUC, Area Under the Curve.

Moreover, the minutes of weekly total PA was further divided into 4 groups (0 minutes, 1–149 minutes, 150–299 minutes, and ≥ 300 minutes) to investigate the possible dose-response relationships between various PA domains and the prevalence of cancer (**Fig 3**). After adjusting for covariates, the model shows that there was a significant linear trend (p = 0.004) between total PA and cancer, but not between any PA subdomains and cancer. The inverse association between total PA and cancer was not significant until the weekly time spent on total PA reached twice or more of the minimum recommended weekly minutes, in short, ≥ 300 minutes [0.68 (0.54, 0.86)]. Furthermore, a predictive model was developed aimed to evaluate the likelihood of cancer occurrence based on the weekly minutes of total PA. As shown in the ROC curve (**Fig 4**), the Area Under the Curve (AUC) was 0.71 (95%CI,0.69–0.73). Sensitivity was 0.67 and specificity was 0.66.

## 4. Discussion

This cross-sectional study investigated the association between weekly minutes spent in various PA domains and the prevalence of cancer among 8963 participants aged 20 to 64 years. The choice to focus on this age group was due to their higher engagement in OPA and TPA, which are key components of this study. After adjusting for age, gender, BMI, race, education level, marital status, smoking, and alcohol consumption, meeting the minimum requirement of the PA guidelines for total PA (≥ 150 minutes per week) was significantly associated with the decreased likelihood of cancer. Although further stratification analysis showed that this association was significant only when the amount of total PA reached twice the minimum recommended amount (≥300 minutes), this might be due to the fact that the number of participants who performed 1–2 times (150–299 minutes per week) the minimum recommended level of PA guidelines was small, 819 of them (about 9.14%). Overall, the participants achieving twice the minimum recommended PA guidelines (≥300 minutes) for total PA were 32% [0.68 (0.54, 0.86)] less likely to be associated with cancer. In addition, the trend analysis revealed that the prevalence of cancer statistically decreased with the increase in total PA amount. Moreover, an AUC of 0.71 suggests that the constructed prediction model has an acceptable predictive ability for cancer occurrence based on weekly minutes of PA.

Consistent with the findings in this study, an increased lifetime average total PA was significantly associated with reduced postmenopausal breast cancer risk, which was reported in a few studies [17–19]. A similar association between pancreas cancer was stated in a meta-analysis of 28 studies [20]. Additionally, not only the link between total PA and cancers but also the dose-response relationships between total PA, measured in metabolic equivalent of task (MET) minutes per week, and cancers were reported [21]. Compared to participants who did not meet the minimum WHO recommended PA level (600 MET minutes/per week) for total PA, the risk of breast cancer reduced by 3%, 6%, and 14% in those who performed about 1–6.7 times (600–3999 MET minutes/per week), 6.7–13.3 times (4000–7999 MET minutes), and ≥ 13.3 times (≥8000 MET minutes) the minimum WHO recommended PA level; the risk of colon cancer reduced by 10%, 17%, and 21% in those who performed about 1–6.7 times, 6.7–13.3 times, and ≥ 13.3 times the minimum WHO recommended PA level [21].

However, significant associations between three PA subdomains (OPA, TPA, and LTPA) and cancers were not found in this study. This finding aligns with previous research conducted by Yonsei University, which similarly reported no significant association between any of these three PA subdomains and colorectal cancer [22]. Conversely, some studies had different findings. A recent study reported that achieving 7.5–15 MET hours per week of LTPA is strongly associated with a reduced risk of seven types of cancer, including colon, breast, endometrial, kidney, myeloma, liver, and non-Hodgkin lymphoma [24]. Another study also showed that women engaged in high levels of OPA were statistically associated with a decreased risk of breast cancer (HR 0.72; 95% CI 0.52–0.98) [23]. Nevertheless, these studies did not consider other PA subdomains but only focused on a single PA subdomain. In other words, the potential interactions introduced by other PA subdomains were not taken into account.

Another finding of this study was that the protective effect of total PA against cancer was significant in participants with a BMI of 18.5–24.9 kg/m2 and 25–29.9 kg/m but not significant in those with a BMI < 18.5 or ≥ 30 kg/m2. Individuals falling into underweight, or obese categories could benefit from meeting the minimum recommended PA guidelines for total PA, but the protective effect of PA is less pronounced. Underweight individuals may have compromised immune function or nutritional deficiencies [32], while immune dysfunction is also in obese individuals and obesity is a significant risk factor for various types of cancer [32, 33]. PA may not be sufficient to counteract the heightened cancer risk associated with underweight and obesity. Similarly, the protective effect of total PA against cancer was significant in populations with an education level of 9-11^th^ grade and college and above, but not significant in those with an education level of less than 9^th^ grade and high school graduates. Previous studies have also shown that education may be related to cancer prevalence, and it stated that low education (< high school) can increase the risk of some cancers, such as bladder cancer in men and colon cancer in women, while it can be associated with a decreased risk of local prostate cancer in men and invasive breast cancer in women [34]. This may be attributed to many other factors, as education may serve as an indicator reflecting individual social class, socioeconomic status, and even occupation and lifestyle to some extent [34]. Addressing lifestyle disparities, such as smoking rate, is considered a practical approach to mitigating cancer disparities related to socioeconomic status [34].

In this study, the association between not only total PA but also various PA subdomains (OPA, TPA, and LTPA) and cancer has been investigated. Previous studies investigated the association between a single PA subdomain but no other subdomains and cancer, so possible interactions among different PA subdomains could be ignored. This study, showing that total PA but not any single PA subdomain is significantly associated with cancer, is trying to respond to this gap. Given the findings, clinical practice should focus on promoting comprehensive PA that includes OPA, TPA, and LTPA to achieve a well-rounded total PA. Healthcare providers should advise patients to aim for at least 150 minutes of total PA per week, with a goal of reaching 300 minutes per week for optimal cancer prevention. Counseling should emphasize the importance of balancing different types of PAs and integrating them into daily life. Healthcare professionals should develop individualized PA plans tailored to patients’ lifestyles and preferences, monitor progress regularly, and offer ongoing support and encouragement. Educating patients about the broad health benefits of total PA, including its potential to reduce cancer risk, is crucial to motivating sustained engagement in diverse PA.

However, there are several limitations of this study. This study’s cross-sectional design allows to examine the associations between PA domains and cancer, but it limits the ability to infer causality. Longitudinal studies are needed to establish temporal relationships and causative links. Moreover, the primary variables of interest were obtained through self- or proxy-reported questionnaires instead of objective measurements, and the amount of every PA subdomain was assessed for a typical week at a single time. Furthermore, only a small group of participants (429) were cancer survivors, so they were not divided into subgroups based on cancer types to assess the association between PA and various types of cancer. Moreover, the interactions between these three PA subdomains need to be further studied.

## 5. Conclusion

This cross-sectional study utilizing data from the NHANES spanning the years 2007 to 2018, discovered a reverse association between total PA and cancer. Specifically, there is no significant association between any of these three single PA subdomains and cancer prevalence other than total PA. Therefore, this study recommends clinical practice should prioritize promoting comprehensive PA that integrates OPA, TPA, and LTPA to achieve at least 150 minutes per week (i.e. per seven days) initially and progressing towards 300 minutes for optimal cancer prevention. Moving forward, future research should explore the synergistic effects of multiple PA subdomains and their long-term impacts on cancer outcomes, thereby informing more effective preventive strategies and interventions in oncological care.
